# PTTG1-interacting protein (PTTG1IP/PBF) predicts breast cancer survival

**DOI:** 10.1186/s12885-017-3694-6

**Published:** 2017-10-27

**Authors:** Heli Repo, Natalia Gurvits, Eliisa Löyttyniemi, Marjukka Nykänen, Minnamaija Lintunen, Henna Karra, Samu Kurki, Teijo Kuopio, Kati Talvinen, Mirva Söderström, Pauliina Kronqvist

**Affiliations:** 1 Department of Pathology and Forensic Medicine, University of Turku and Turku University Hospital, Kiinamyllynkatu 10, 20510 Turku, Finland; 2 Department of Medical Statistics, Medical Faculty, University of Turku, Lemminkäisenkatu 1, 20510 Turku, Finland; 3 Biological and Environmental Science, University of Jyväskylä and Department of Pathology, Central Finland Health Care District, Keskussairaalantie 19, 40620 Jyväskylä, Finland; 4 Department of Pathology, Pori Central Hospital, Sairaalantie 3, 28500 Pori, Finland; 5 Auria Biobank, University of Turku and Turku University Hospital, Kiinamyllynkatu 10, 20510 Turku, Finland

**Keywords:** PTTG1IP, PBF, Immunohistochemistry, Breast cancer, Prognosis

## Abstract

**Background:**

PTTG1-interacting protein (PTTG1IP) is an oncogenic protein, which participates in metaphase-anaphase transition of the cell cycle through activation of securin (PTTG1). PTTG1IP promotes the shift of securin from the cell cytoplasm to the nucleus, allowing the interaction between separase and securin. PTTG1IP overexpression has been previously observed in malignant disease, e.g. in breast carcinoma. However, the prognostic value of PTTG1IP in breast carcinoma patients has not previously been revealed.

**Methods:**

A total of 497 breast carcinoma patients with up to 22-year follow-up were analysed for PTTG1IP and securin immunoexpression. The results were evaluated for correlations with the clinical prognosticators and patient survival.

**Results:**

In our material, negative PTTG1IP immunoexpression predicted a 1.5-fold risk of breast cancer death (*p* = 0.02). However, adding securin immunoexpression to the analysis indicated an even stronger and independent prognostic power in the patient material (HR = 2.5, *p* < 0.0001). The subcellular location of securin was found with potential prognostic value also among the triple-negative breast carcinomas (*n* = 96, *p* = 0.052).

**Conclusions:**

PTTG1IP-negativity alone and in combination with high securin immunoexpression indicates a high risk of breast cancer death, resulting in up to 14-year survival difference in our material.

## Background

Pituitary tumour transforming gene 1 interacting protein (PTTG1IP, also PTTG1-binding factor, PBF) is an oncogene, originally detected as a 22 kDa protein binding to securin [1, 2]. Securin, the protein product of *PTTG1*, in turn, is a proto-oncogene first described in rat pituitary tumour cells [3]. Both proteins are key actors in the complex regulatory network responsible for the timed and controlled release of the sister chromatids during the metaphase/anaphase transition of the cell cycle (Reviewed in [4]).

Increasing evidence suggests that the subcellular localizations of both PTTG1IP and securin are critical for the progression of mitosis in metaphase-anaphase transition. PTTG1IP promotes the activation of securin by facilitating the shift of securin from the cell cytoplasm to the nucleus, allowing the interaction between separase and securin [1]. In addition to its role in metaphase/anaphase transition, PTTG1IP has been reported to interact with securin in the transactivation of FGF-2 (fibroblast growth factor 2) [1] and in the regulation of the human sodium symporter in thyroid cells [5]. However, to this day, the full range of the PTTG1IP function is yet unrevealed.

PTTG1IP has been shown ubiquitously expressed in several human tissues, such as placenta, thyroid gland, lymph node and bone marrow [1]. As uncontrolled sister chromatid separation is one of the hallmarks of malignant progression, it is expectable that PTTG1IP overexpression has previously been observed in malignancy, eg. in thyroid [6], breast [7] and colorectal carcinoma [8]. However, the prognostic value of PTTG1IP in breast carcinoma patients has not previously been revealed.

In previous literature, little has been published about the immunohistochemical expression of PTTG1IP in human malignancies. Hsueh et al. [6] have demonstrated that compared to normal thyroid tissue, PTTG1IP is highly expressed in 61.4% of papillary thyroid carcinomas and that high PTTG1IP expression is correlated with a shorter disease-specific survival time. To our knowledge, no previous studies have evaluated PTTG1IP as a prognostic biomarker for breast carcinoma patients. We have previously presented that the lack of PTTG1IP in breast cancer cells is associated with cytoplasmic localization of securin and that this protein expression profile was overrepresented in the triple-negative breast cancers (TNBC) [9]. In the present paper, we report on the expression pattern of PTTG1IP in 497 breast carcinomas, with up to 22 years of follow-up. Included is also a separate material of 96 triple-negative breast carcinomas. As analysed in a material comprising of all breast cancer subtypes (*n* = 401), breast carcinomas lacking PTTG1IP immunoexpression and particularly the lack of PTTG1IP expression in combination with high securin expression were associated with an increased risk of breast cancer death. The analyses were repeated in a separate material of triple negative breast carcinomas, where we could verify that the cytoplasmic as opposed to the nuclear localization of securin in the cancer cells indicated a survival difference among the patients, a finding providing support to the interpretation that PTTG1IP participates in the regulation of securin activity by controlling its subcellular location.

## Methods

### Patients and tissues

The patient material comprises unilateral, primary breast carcinomas of a total of 497 female patients. A larger part of the material, a total of 401 patients with breast carcinomas, consisted of all subtypes and were diagnosed and treated during 1987 – 1997 in Central Finland Central Hospital, Jyväskylä, Finland. A smaller part of the material, a total of 96 patients, represented a separate set of triple-negative breast carcinomas (TNBCs) diagnosed and treated in Turku University Hospital during 2005-2015 and obtained from Auria Biobank, Turku, Finland (Table 1).

**Table 1:** Summary of the patient and tissue materials with clinico-pathologic characteristics of the breast carcinomas

		All subtypes	TNBC
No. of patients			
	All carcinomas	401	
	Triple-negative carcinomas	47	96
Patient characteristics			
	Mean age at diagnosis (range) (years)	56 (39 - 78)	62 (32-93)
	Axillary lymph node positive (%)	187 (46%)	33 (35%)
	Mean tumor size (range) (cm)	2.4 (0.2-10.0)	2.7 (0.8-18.0)
	Histological type (%)		
	Infiltrating ductal	332 (82%)	96 (100%)
	Special type	72 (18%)	0
	Intrinsic subtype (%)		
	Luminal	281 (69%)	0
	Her2-amplified	51 (15%)	0
	Triple-negative	47 (17%)	96 (100%)
	Breast cancer deaths (%)	141 (35%)	21 (22%)

The table summarizes the two patient materials applied, the first one consisting of all breast cancer subtypes of the intrinsic classification and the second one representing triple negative breast carcinomas (TNBC)

All patients were treated with surgical resection or mastectomy with a sentinel node biopsy and, in case of metastatic disease, axillary evacuation. Postoperative radiation therapy and adjuvant treatments were applied according to the international guidelines for breast cancer classification and treatment at the time of the diagnosis [10]. No preoperative adjuvant treatments were administered. Complete clinical follow-up information was available from pathology reports, patient files of all patients and Auria Biobank. The follow-up data included the established prognosticators of clinical breast cancer treatment, i.e. axillary lymph node status, tumour size, histological grade, hormone receptor and *HER2*-oncogene status, proliferation marker Ki-67, and intrinsic classification defined according to the surrogate guidelines by the 12th St Gallen International Breast Cancer Conference (2011) Expert Panel [11]. Causes of death were obtained from autopsy reports, death certificates and from the Finnish Cancer Registry. The maximum follow-up periods for prognostic associations were 22 years and 6 months (mean 10.0 years) for the larger material and 11 years and 9 months (mean 5.1 years) for the smaller material.

All tissue materials were prepared according to the standard practice of a clinical histopathology laboratory, i.e. fixed in buffered formalin (pH 7.0) and embedded in paraffin. Immunohistochemistry was performed on tissue microarrays (TMAs) comprising two tissue cores from the tumour of each patient. The TMAs were prepared by punching the paraffin block of each tumour with either a 0.6 mm (all breast carcinoma subtypes) or a 1.5 mm diameter cylinder (TBNCs).

### Immunohistochemistry*,* in situ hybridization and immunofluorescence

The immunohistochemical (IHC) staining of PTTG1IP was performed with rabbit polyclonal anti-PTTG1IP antibody (Abcam, Cambridge, UK, ab128040) on an automated immunostaining Discovery XT machine (Roche Diagnostics/Ventana Medical Systems, Tucson, AZ, USA). Deparaffinization, epitope retrieval (CC2 20 min) and primary antibody incubation (20 min at 37 C, dilution 1:500) were done before detection with OmniMap HRP and ChromoMap DAB kits (Roche/Ventana). Securin IHC was performed with mouse monoclonal antibody (Abcam, ab3305, clone DCS-280, dilution 1:100) using Lab Vision Autostainer 480 (Thermo-Fisher Scientific, Fremont, CA, USA) and detection with PowerVision + polymer kit, according to standard protocol (DPVB + 110HRP; Immunovision Technologies, Vision Biosystems, Norwell, MA, USA). For oestrogen receptor (ER), progesterone receptor (PR), Ki-67 and HER2 IHC, an automated immunostaining BenchMark XT machine (Roche Diagnostics / Ventana) and an ultraView Universal DAB Detection Kit (Roche/Ventana) were used as described previously [9, 12]. Initial HER2 testing was performed on basis of immunopositivity according to international guidelines and specimen representing 2+ and 3+ staining patterns were allocated for further verification [13].

In situ hybridization (ISH) was applied for the final confirmation of the *HER2*-amplification status. *HER2*/*Chr17* double in situ hybridizations were performed using BenchMark XT machine (Roche diagnostis /Ventana), the HER2 DNA and the Inform Chromosome 17 probe set, and the ultraView SISH detection kit to detect *HER2* (Roche/Ventana) and the ultraView Alkaline Phosphatase Red ISH Detection Kit to detect *Chr17* (all from Roche Diagnostics /Ventana) (Roche/ Ventana).

For the PTTG1IP and securin double immunofluorescence (IF), sections were stained manually using a tyramide signal amplification system for the sequential detection of rabbit and mouse primary antibodies (TSA ™Kits #41 and #2 with Alexa Fluor® 555 and 488 tyramides, Molecular Probes. LifeTechnologies, Eugene, OR, USA). More detailed description of the double staining has been published previously [9].

### Interpretation of IHC

**Fig. 1:**
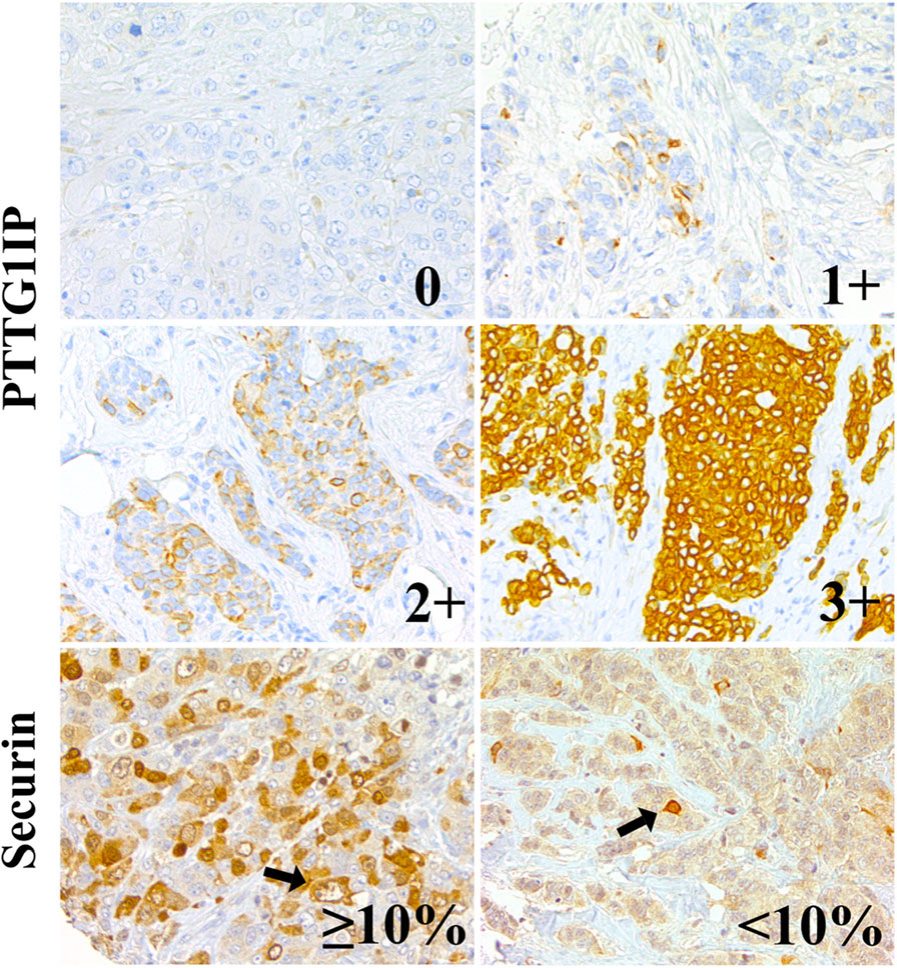
Examples of immunoexpression patterns of PTTG1IP and securin in breast carcinomas. PTTG1IP was observed as a diffuse cytoplasmic staining, and scored based on the extent of positive cancer cells as negative (score 0) and mild to strong positivity (scores 1+ − 3+). Securin was observed both in the nucleus and cytoplasm of the cancer cells and classified according to the fraction of positively-staining cancer cells (<10% vs. ≥10% of cancer cells). Examples of nuclear and cytoplasmic staining patterns are indicated with arrows. (200× magnification)

In the cancer cells PTTG1IP immunopositivity was observed as a diffuse cytoplasmic staining (Fig. 1). Occasional faint or granular cytoplasmic positivity was observed but these cells were interpreted as negative. Tissue cores with less than 100 cells were excluded from the study. The intensity of the staining was found uniform over the cases and, therefore, intensity was not registered in the evaluations. In benign breast epithelium, only single positive cells were detected.

Based on the extent of the observed diffuse cytoplasmic staining in cancer cells, the breast cancer cases were divided into four subgroups. The cases showing no PTTG1IP expression were scored as 0. The cases with less than 10% PTTG1IP-positive cells were scored as 1, the cases with 10-50% immunopositive cancer cells were scored as 2 and the cases showing more than 50% immunopositive cancer cells as 3.

The evaluation of the PTTG1IP-immunopositivities was performed by a single histopathologist (HR for the all subtypes material and PK for the TNBC material). Prior to the evaluations, the consistency of the interpretation was verified by using repeated evaluations by a single observer (HR) or independent evaluations by two observers (HR and PK) in a set of 81 breast cancer tissue cores. The resulting intra- and interclass reproducibility (PTTG1IP negative vs. positive) was 0.961 and 0.881, correspondingly.

Immunostaining pattern for securin was observed as a combination of nuclear and cytoplasmic expression as previously described by Karra et al. [12] (Fig. 1). In each carcinoma case, the predominant subcellular localization of securin observed in >90% of cancer cells was registered as previously described [9].

### Statistical analysis

In statistical analysis, patients were allocated into different expression groups based on the observed level of PTTG1IP-immunopositivity. The different PTTG1IP expression levels (0-3) were tested in univariate analyses and it was noticed that categorization at 0 (negative) vs 1-3 (mild to strong positivity) produced the strongest predictive value of disease survival in our material. This categorization was also supported by morphological observations of PTTG1IP immunohistochemistry in the breast cancer material and by the associated very high consistency of interpretations as calculated inter- and intraclass reproducibility. Categorization of securin immunoexpression was performed on the basis of the fraction of immunopositive cancer cells (<10% vs. ≥10%) and subcellular location (>90% of cancer cells showing cytoplasmic vs. nuclear expression) as described previously [9, 12].

Associations between PTTG1IP and securin were analysed by Fisher’s exact test. Survival analyses were first started with univariate analysis for time to breast cancer-specific death, estimated using the Kaplan-Meier technique. Thereafter, the differences between categorized values were tested using the Wilcoxon test. For further analysis involving more complicated models with multiple factors, we evaluated the studied immunoexpressions of PTTG1IP and securin in addition to the established prognosticators of breast cancer i.e. nodal status, tumour size, histological grading, intrinsic classification and proliferation as expressed in Ki-67- immunopositivity. Qualification for multivariate analysis in the two separate patient materials was based on the observed statistical significance in univariate settings. The final analyses were performed with the help Cox proportional hazard models. Differences between categories were quantified by calculating hazard ratios (HRs) with 95% confidence intervals (95 CIs). Patients with missing data were automatically excluded from the analyses. *P*-values less than 0.05 (two-tailed) were considered statistically significant. The statistical computations were performed using SAS System for Windows, version 9.3 and 9.4 (SAS Institute Inc., Cary, NC, USA).

## Results

**Fig. 2:**
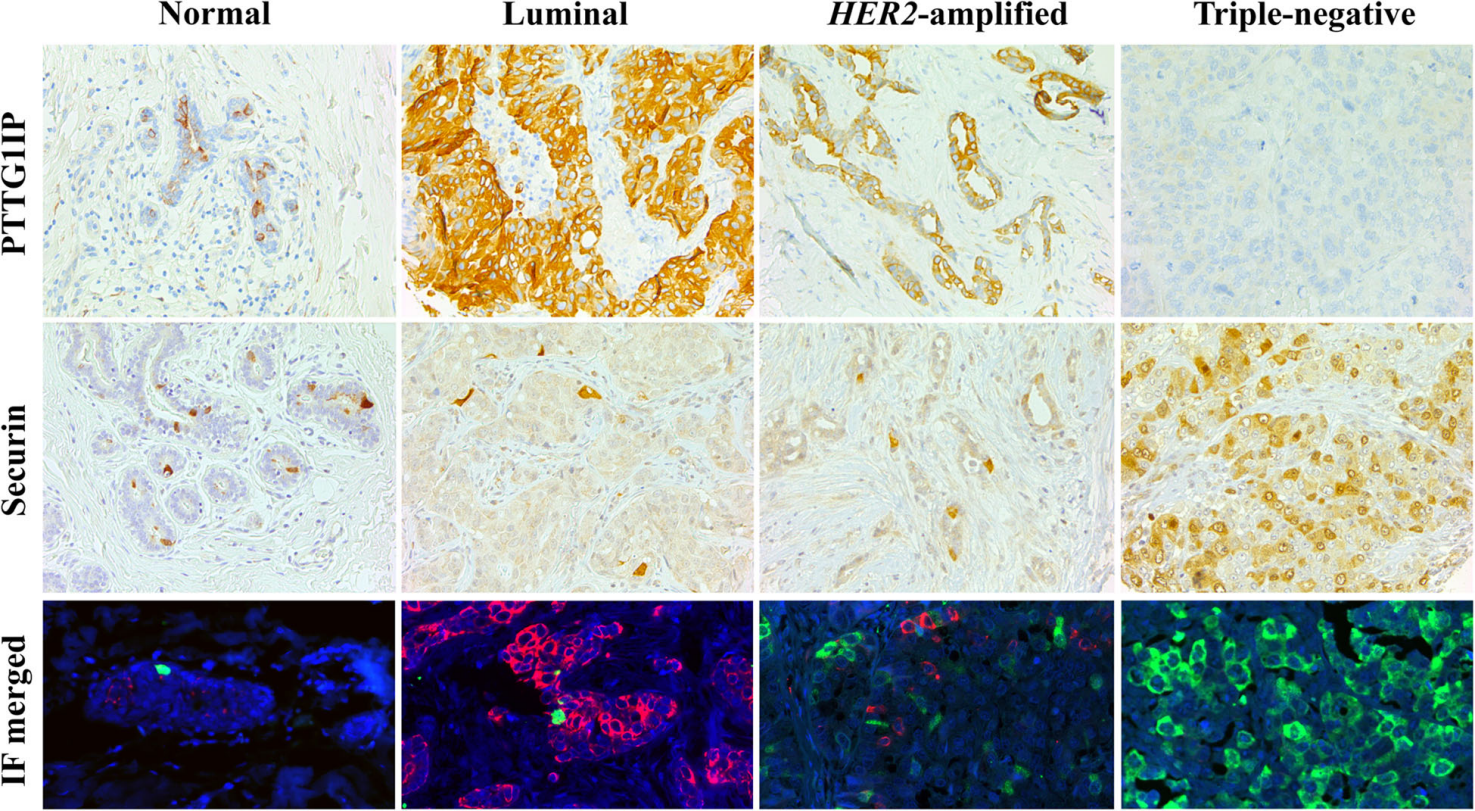
Photomicrographs of PTTG1IP and securin immunoexpression in benign breast epithelium and in breast carcinomas representing luminal, HER2-amplified and triple-negative subtypes. IF double staining demonstrates the co-localization of PTTG1IP and securin in benign and malignant epithelium (red signal PTTG1IP and green signal securin). (200× magnification)

The patterns of immunostaining in benign breast epithelium and in different types of invasive breast carcinomas are demonstrated in Fig. 2. In benign breast epithelium, only single epithelial cells showed PTTG1IP-positivity. Among breast carcinomas of all subtypes (*n* = 401) PTTG1IP-immunopositivity was seen in 74.3% of the cases. Evaluating the extent of PTTG1IP-positivity revealed that 17% of carcinomas showed weak staining (<10% of cancer cells, score 1+), 41% moderate staining (10-50% of cancer cells, score 2+) and 15% diffuse staining (≥50% of cancer cells, score 3+). Total PTTG1IP-negativity (score 0) was observed in 26% of carcinomas. In the TNBCs (*n* = 96), 55 of the cases were PTTG1IP negative (57%). No PTTG1IP expression was observed in the stromal fibroblasts or inflammatory cells. As for securin, the extent and the subcellular locations of immunoexpression patterns in benign and malignant breast epithelium were observed as previously reported [12] (Fig. 2).

Table 2 represents the distribution of PTTG1IP and securin expressions in subgroups divided according to the established prognosticators of breast cancer i.e. tumour size, axillary lymph node metastasis, histological grade and intrinsic classification. In all subgroups of the established prognosticators, PTTG1IP-immunopositivity was inversely related to the aggressiveness of the disease (*p* < 0.001). Instead, high expression and cytoplasmic localization of securin was directly associated with aggressive disease features (*p* < 0.001). A significant association between the PTTG1IP expression and the subcellular location of securin could be observed (*p* < 0.001). This statistical association could also be verified morphologically in IF-double-staining where cancer cells exhibiting PTTG1IP-positivity showed nuclear securin expression while, in absence of PTTG1IP, securin expression showed a shift towards cytoplasmic subcellular location (Fig. 2).

**Table 2:** PTTG1IP and securin in breast carcinoma

	All subtypes			TNBC		
	n=401			n=96		
	Neg PTTG1IP (%)	High securin (%)	Cytoplasmic securin (%)	Neg PTTG1IP (%)	High securin (%)	Cytoplasmic securin (%)
All patients	25.7	39.5	17.5	57.3	82.6	79.2
Nodal status						
Node -	22.3	43.4	7.9	38.5	36.5	47.9
Node +	27.1	63.1	9.2	15.6	21.9	30.2
Tumour size						
<2cm	18.2	19.4	4.7	25.0	21.9	29.2
≥2cm	28.6	35.6	13.1	29.2	34.4	47.9
Histological grade						
I	8.8	11.5	1.7	0	0	0
II	21.5	26.1	14.1	0	0	0
III	49.4	64.2	25.2	100	100	100
Intrinsic classification						
Luminal	11.5	29.6	3.2			
HER2-amplified	25.5	40.7	8.7			
Triple-negative	63.8	62.7	11.9			

The table summarizes for each patient group the fractions (%) of cases with immunoexpression patterns indicating aggressive course of disease (negative PTTG1IP, high securin expression^a^ and cytoplasmic securin expression). The results are presented separately for all subtypes of breast carcinomas (n=401) and for TNBCs (n=96) and in subgroups divided according to the established prognosticators of breast cancer

^a^High securin expression: securin-positivity observed in ≥10% of cancer cells

**Fig. 3:**
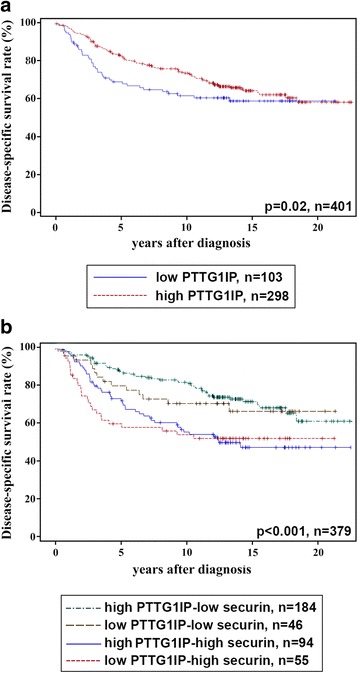
Kaplan-Meier analysis of breast cancer survival based on all breast cancer subtypes (*n* = 401) demonstrating survival difference associated with PTTG1IP immunoexpression (**a**) and the combination of the immunoexpressions of PTTG1IP and securin (**b**)

In prognostic analyses, the breast cancer cases were analysed as divided into two categories as described above (PTTG1IP 0 vs. 1+ − 3+ and securin <10% vs. ≥10% of cancer cells). In univariate analysis involving all breast cancer subtypes, PTTG1IP-negativity predicted a 1.5-fold risk for breast cancer death (*p* = 0.002, CI 0.9-2.7). The different survivals of PTTG1IP-negative and PTTG1IP-positive subgroups of patients are demonstrated in Kaplan-Meier curves (Fig 3a). Further quartile estimations of survival curves indicated that the majority (75%) of the patients with PTTG1IP-positive carcinoma survived 11.4 years while the majority of patients a carcinoma lacking PTTG1IP expression were alive only 6.4 years after diagnosis. Securin showed an even stronger prognostic value both for all subtypes of breast carcinomas as evaluated based on the extent (*p* < 0.0001, HR 2.7 and CI 1.6-2.7) and the subcellular localization of immunoexpression (*p* = 0.003, HR 1.6, CI 1.1-2.4). A particularly convincing survival difference was observed for patients of all breast cancer subtypes between the most favourable (high PTTG1IP and low securin) and the most unfavourable (low PTTG1IP and high securin) outcome of the disease (Fig. 3b). In quartile estimations of the Kaplan-Meier analysis, the majority (75%) of patients showing the most favourable combination of PTTG1IP and securin survived 17.6 years after diagnosis as opposed to 3.5 years among patients with unfavourable combination of the studied proteins.

When the analysis of PTTG1IP and the subcellular location of securin was repeated in a separate material consisting of TNBCs, it sparsely failed to show statistical significance. The cases exhibiting cytoplasmic securin were associated with a 5.7-fold risk of breast cancer death as compared to cases with a nuclear localization of securin immunoexpression (*p* = 0.052, CI 0.8-42). The patients with cytoplasmic securin expression had an estimated 5-year breast cancer-specific survival rate of 75%, as compared to 95% among the patients with nuclear securin expression. The expression of PTTG1IP or extent of securin expression did not show statistically significant prognostic associations among the TNBCs, possibly due to the small number of patients or the shorter follow-up period in the material.

Multivariate analysis was performed for PTTG1IP and securin, and the established prognosticators of breast cancer among all subtypes of breast carcinomas (*n* = 401). As the results, the significant independent prognosticators of disease-specific survival were only nodal status, tumour size and securin (Table 3). PTTG1IP expression did not show prognostic value in the multivariate setting.

**Table 3:** Multivariate analyses for PTTG1IP and securin immunohistochemistry, and established prognosticators^a^

	HR	P	CI
PTTG1IP		NS.	
Securin	2.5	<0.001	1.5-4.1
Tumour size	1.3	0.001	1.1-1.5
Nodal status	2.6	<0.001	1.6-4.1
Histological grade			
grade 1 vs 2-3		NS.	
grade 3 vs 1-2		NS.	
Intrinsic classification			
Luminal vs others		NS.	
Triple─ vs others		NS.	

*Abbreviations*: *HR* Hazard ratio, *P* P-value of Wilcoxon's rank sum test, CI = 95% confidence interval, *NS* No statistical significance

The analysis is based on 401 breast carcinoma patients with up to 22-year follow-up

^a^Analysis has been performed on material divided into subgroups with favourable vs

unfavourable prognosis as follows:

PTTG1IP stratified into positive vs negative immunoexpression

Securin stratified into low vs high immunoexpression (<10% vs ≥10% positive cancer cells)

Tumour size stratified into small vs large (≤2cm vs >2cm in diameter)

Nodal status stratified into axillary lymph node negative vs positive

Histological grade stratified into grades 1, 2 and 3

Intrinsic classification stratified into luminal, *Her2*-amplified and triple-negative

## Discussion

In the literature, PTTG1IP has been connected with multiple pathways promoting malignancy. Among them, observations from breast cancer and colorectal carcinoma cell cultures have indicated that the overexpression of PTTG1IP increases cell invasion independent of increased proliferation [7, 8]. Evidence from papillary and anaplastic thyroid carcinoma cell cultures and human colorectal carcinoma suggests that the binding of PTTG1IP to p53 dysregulates the p53 function [8, 14]. Previously, PTTG1IP overexpression has been associated with a poor prognosis in thyroid [6, 14] and colorectal carcinomas [8].

In breast carcinoma, an association between ER and PTTG1IP has been reported. In normal breast epithelium, the expression of PTTG1IP is low and limited to single cells but in ER positive breast cancer PTTG1IP is overexpressed in both cell cultures and in tissue samples [7]. This may be explained by the structure of the PTTG1IP promoter, which has an oestrogen response element (ERE) facilitating its regulation by activated oestrogen receptor alpha (ERα). Also, exposing breast cancer cell cultures to oestrogen has been shown to up-regulate PTTG1IP expression [7]. It has also been reported that the variable number of tandem repeats in PTTG1IP promoter might be associated with an increased risk of ER-positive breast cancer [15]. Very few previous reports have correlated the findings with the immunohistochemical expression of PTTG1IP. Also, little has been previously known about the expression of PTTG1IP in ER-negative breast carcinoma.

In the present paper, we report on the expression patterns and prognostic value of PTTG1IP-immunoexpression in a total of 497 human breast carcinomas, including 96 cases of the triple-negative subtype. The main part of our results supports the previous literature on PTTG1IP overexpression in breast carcinoma. In contrast to previous literature [6, 8, 14], in our material comprising all breast carcinoma subtypes and up to 22-year follow-up, it was PTTG1IP-negativity that was associated with a 1.5-fold risk of breast cancer death. This PTTG1IP negative subgroup comprised the majority (78%) of triple-negative breast carcinomas where negative PTTG1IP expression was combined with high securin expression located in the cytoplasm of the cancer cells. This finding is in line with previous literature indicating the lack of PTTG1IP restricts the nuclearization of securin [1, 9]. However, a statistically significant prognostic association for PTTG1IP-negativity could not be shown in the TNBC material, possibly due to small number of patients (*n* = 96). Still, our results highlight the superior prognostic value of high securin expression both alone and in combination with PTTG1IP. Interestingly, among triple-negative breast carcinomas only the subcellular localization of securin in the cytoplasm of cancer cells was associated with prognostic value although also this association sparsely failed to show statistical significance (*p* = 0.052).

The pathways leading to PTTG1IP regulation are still unsettled but miRNA-584, a known tumour suppressor, has been suggested among them. In glioma cells, miRNA-584-expression has been shown to result in PTTG1IP down-regulation and suppression of tumour cell proliferation while down-regulation of miRNA-584 has been observed to up-regulate PTTG1IP and increase proliferation [16]. In breast cancer cell lines, TGF-β induced downregulation of miRNA-584 has been suggested as one of the crucial steps for cell migration [16].

Also, hepatitis virus B has been shown to induce miRNA-122 down-regulation leading to overexpression of PTTG1IP, and both an increase in cell proliferation and invasion in hepatocellular carcinoma cell lines [17]. Although some mutations in the *PTTG1IP* gene can be found listed in databases such as the COSMIC (Catalogue of Somatic Mutations in Cancer) [18], the clinical relevance of these mutations is still unclear. In our TNBC material, no mutations affecting the protein expression and function of *PTTG1IP* could be found (data not shown).

Recently, PTTG1IP has been implied in cancer treatment. Previously, PTTG1IP-overexpression has been shown to repress the effect of radioiodide treatment in thyroid carcinoma [19]. More recently, Smith and co-workers [20] have, however, suggested that radioiodide uptake in thyroid cancer cells could be increased and, thus, efficacy of the treatment could be improved by targeting PTTG1IP expression via the modulation of phosphorylation. Interestingly, similar effect of regulation of PTTG1IP expression by phosphorylation has been observed in breast cancer cells [20]. Moreover, decreased cell growth has been demonstrated in HeLa cells when securin expression has been suppressed by a PTTG1IP combined with the F-box motive of β-TrCP [21].

## Conclusions

PTTG1IP has an important role in the complex regulatory network responsible for the metaphase/anaphase transition of the cell cycle. We have demonstrated that the lack of PTTG1IP expression alone and in combination with high and cytoplasmic securin expression is associated with an increased risk of breast cancer death. More research is required to further examine this association in TNBCs. In previous literature, modulation of PTTG1IP expression has already been investigated and appears a promising pathway for development of future cancer treatments.

## Abbreviation

ER: Oestrogen receptor
FGF-2: Fibroblast growth factor 2
HER2: Human epidermal growth factor 2
IF: Immunofluorescence
PBF: Pituitary tumour transforming gene 1 binding factor
PR: Progesterone receptor
PTTG1: Pituitary tumour transforming gene 1
PTTG1IP: Pituitary tumour transforming gene 1 interacting protein
TMA: Tissue micro array
TNBC: Triple negative breast cancer
